# Visualization of the Research Panorama of Decision-Making in Soccer: Bibliometric Analysis with VOSviewer and Review of the Most Cited Studies of the Last 15 Years (2010–2024)

**DOI:** 10.3390/sports13060177

**Published:** 2025-06-03

**Authors:** Juan David Paucar Uribe, Boryi A. Becerra-Patiño, Jorge Olivares-Arancibia, Rodrigo Yáñez-Sepúlveda, Aldo Vasquez-Bonilla, Daniel Rojas-Valverde, José Francisco López-Gil, Guilherme Machado

**Affiliations:** 1 Faculty of Physical Education, National Pedagogical University, Valmaria Cl. 183 # 5199, Bogotá 111166, Colombia; jdpaucaru@upn.edu.co (J.D.P.U.); or boryialexander.becerrap@um.es (B.A.B.-P.); 2 Programa de Doctorado en Ciencias de la Actividad Física y del Deporte, University of Murcia, 30720 Santiago de la Ribera, Spain; 3 Grupo AFySE, Investigación en Actividad Física y Salud Escolar, Escuela de Educación Física, Facultad de Educación, Universidad de las Américas, Santiago 7500975, Chile; jorge.olivares.ar@gmail.com; 4 Facultad de Educación y Ciencias Sociales, Universidad Andres Bello, Viña del Mar 2520000, Chile; rodrigoyanezs@unab.cl; 5 Grupo de Avances en Entrenamiento Deportivo y Acondicionamiento Físico, Facultad de Ciencias del Deporte, Universidad de Extremadura, 10003 Cáceres, Spain; alvasquezb@unex.es; 6 Centro de Investigación y Diagnóstico en Salud y Deporte (CIDISAD-NARS), Clínica de Lesiones Deportivas (Rehab&Readapt), Escuela Ciencias del Movimiento Humano y Calidad Vida (CIEMHCAVI), Universidad Nacional de Costa Rica, Heredia 86-3000, Costa Rica; sofia.gonzalez.rojas@est.una.ac.cr; 7 School of Medicine, Universidad Espíritu Santo, Samborondón 092301, Ecuador; 8 Vicerrectoría de Investigación y Postgrado, Universidad de los Lagos, Osorno 5290000, Chile; 9 Scientific Department and Department of Athletes’ Integration and Development, Paulista Football Federation (FPF), São Paulo 05614-060, Brazil; machado.guilhermef@gmail.com; 10 Department of Physical Education, Centre of Research and Studies in Soccer (NUPEF), Universidade Federal de Viçosa, Viçosa 36570-900, Brazil

**Keywords:** assessment, perception, attention, performance, team sport

## Abstract

Background/Objectives: Various studies have investigated the importance of perceptual–cognitive skills in decision-making and the expert performance of athletes. However, bibliometric study has yet to identify research trends on this topic. The objective of this study was to perform a bibliometric review to identify the research trends in the study of soccer decision-making. Method. A total of 172 studies were included in the databases. Results. The year 2021 was the year with the highest number of published studies (*n* = 23), and 2016 was the year with the highest number of citations (*n* = 692). The average number of citations per document was 19.79. The concepts that have the greatest occurrence in the investigations are performance (*n* = 68), decision-making (*n =* 54), expertise (*n =* 32), skill (*n =* 23), and anticipation (*n =* 22). The journals with the highest number of published documents are the *Journal of Sport Sciences* (10 documents and 437 citations) and *PLoS One* (11 documents and 349 citations). The countries with the highest number of published documents and citations are England (*n =* 46 documents and 996 citations), Germany (*n =* 32 documents and 749 citations), and Spain (*n =* 38 documents and 597 citations). German Sport University Cologne is the organization that has the most publications and citations (*n =* 19 and 531). Conclusions. Existing knowledge production on decision-making in soccer has a preference for the study of two major categories: one related to the analysis of the factors associated with perceptual–cognitive skills, mental fatigue, anticipation, creativity, and memory, whereas the second is more related to the study that has decision-making in the manifestations of specific game performance, between experts and novices, in the precision of technical actions, such as the pass, as well as in a methodology for the selection of athletes.

## 1. Introduction

Overall, sports are characterized by relationships and interactions between athletes [1], which are manifested in the development of game-changing and unpredictable actions [2]. Soccer is a practice where interpersonal and group cooperation are fundamental in the development of sport [3], so understanding individual and collective decision-making could lead to competition [4]. Research in recent decades has made it possible to identify the importance of the perceptual–cognitive skills associated with the expert performance of athletes [5], including the exploration of the environment to understand the kinematic and positional signals [6,7,8], the recognition of the game patterns that are specific to football [9], and the perception and intention of deception toward the opponents that is carried out to favor the actions of play [10,11].

Accordingly, players’ decision-making is affected by different factors, such as the complexity of the task or action to be solved, the time available, and the contextual background available in the various situations of the game [4]. Among them is understanding and tactical understanding [12], where the player must understand, develop, and identify the most significant amount of information to increase effectiveness [13], quickly restoring his attention [14], and, therefore, cognitive effort is not demanding in the interaction of declarative and procedural tactical knowledge during the game [15]. Likewise, the physical demand of athletes produced by accelerations, decelerations, speeds, and rhythms can increase or decrease according to the group organization and individual understanding [16], so consolidating timely decisions in the total load of fatigue avoids injury levels [17] and increases the capacity of effective passes [18], where it is necessary to implement appropriate pedagogical strategies for the development and acquisition of skills [19]. The consolidation of the sports process in children’s and youth stages is vital in developing players’ capacities to carry out actions in decision-making [20]. Therefore, the implementation of didactics, such as games in small spaces, has generated interest in the sports science community for the evaluation and detection of talent [21], increasing scientific production to optimize footballers’ sports performance [22].

On the other hand, understanding soccer as a sport with open skills [23] is subject to complex challenges in mental fatigue [23,24] as a consequence of the different visual stimuli of the unpredictable trajectory of the ball, the opponents, the equipment, and objects, and limiting logic and understanding, which, as a consequence, constantly predisposes athletes to a state of alertness [14], decreasing sports performance, as various investigations have shown [25,26]. The evolution of the sporty human being and the same sport has increased technological advancement. Technology is not usually useful on some occasions; since it can impair performance, an example of this is that the use of social networks before a competition negatively influences the constant decisions of the players [27], where demanding mental loads must be reduced to establish a better relationship in the understanding of the game [28]. In contrast, the behavior in the individual decisions of footballers has allowed for the creation of instruments through video diagnostics that allow for the recognition of specific actions of football [29], such as the TacticUP video test that comprises offensive and defensive actions in football 11 vs. 11 [13] and tests of the speed of reaction in motor development that are used by neuroscience [30]. This allows for the evaluation of different components, such as measurements of dribbling capacity, support for the dribbling player, and the constant relationship between the ball and the player [31].

Other methods, such as artificial intelligence [32] and three-dimensional multiple object tracking (3D-MOT), were created to develop fundamental and attentional dynamic visualizations during sports to perceive relevant information adequately [33]. Their functions have been associated with dynamic environments and decision-making; despite this, these technologies are still under discussion, generating new challenges in the implementation of technologies in football [34]. For their part, they must consider the specificity of the sport and the level of performance of the athletes [35]. Likewise, characterization and individualization by game positions allow us to understand physical differences [36] and technical–tactical actions [37], which are evident in the investigative interest in decision-making in the goalkeeper, in actions of shots to goal [38], and trajectories from the penalty spot [39,40]; however, in the literature, there is no clarity about the differences in decision-making in the other game positions.

The analysis of the performance factors that influence decision-making is associated with the specific restrictions of time, space, and existing relationships with the receiver or adversary to promote the development of perceptual–cognitive skills, such as anticipation and choice of action [41,42], which help improve the execution carried out by the athlete from complex dynamic motor tasks [43]. In another study, developed by McLoughlin et al. [44] through a qualitative approach through the methodology discussion group, which sought to explore decision-making processes in expert players and almost all expert players, it was concluded that decision-making has four critical points: (1) context prior to the game (coach tactics and instructions, opponent level, and importance of the match or competition); (2) current context of the game (scoreboard and playing time); (3) visual information; and (4) individual differences in the athletes (self-efficacy, perceived pressure, morphofunctional characteristics, fatigue). In this sense, fatigue is a key element to consider in the player’s decision-making produced by contextual or environmental variables [4].

With all the above, the scientific evidence continues to focus on different approaches and the consideration of different perceptual–cognitive skills to better understand decision-making. Thus, various studies have concluded that it is necessary to continue developing research that integrates concepts and methodologies from the multiple perspectives with which decision-making has been studied in team sports, such as soccer [4,45]. The same scientific evidence suggests new methodologies to understand the interactions among the different processes that contribute to the development of new research perspectives [46]. These research advances should be put into practice to improve training methods and decision evaluation processes in response to the specificity of the sport and the individuality of the player. To our knowledge, a bibliometric study evaluating decision-making in soccer has not been previously reported in scientific literature. Therefore, the objective of this study was to perform a bibliometric review to identify the trend of the research carried out on decision-making from the use of bibliometrics as a research technique in the Web of Science and PubMed databases, including the documents published between 1 January 2010 and 15 July 2024.

## 2. Materials and Methods

### 2.1. Design

This study was developed using bibliometrics as a research technique. Taking as a reference, the theoretical nature of the study and the classification established by Ato et al. [47], a bibliometric analysis is considered a theoretical and retrospective study because it responds to the purpose of systematizing, analyzing, and condensing a large amount of data related to the temporality of the studies and the evolution of the themes addressed over the years [48]. These types of studies analyze trends that are developed in a thematic area to generalize the production of knowledge through specific criteria such as (i) authors; (ii) countries; (iii) institutions; (iv) keywords; (v) document citations; (vi) citations between cited references; and (vii) editorial groups [49].

### 2.2. Data Extraction

**Figure 1:**
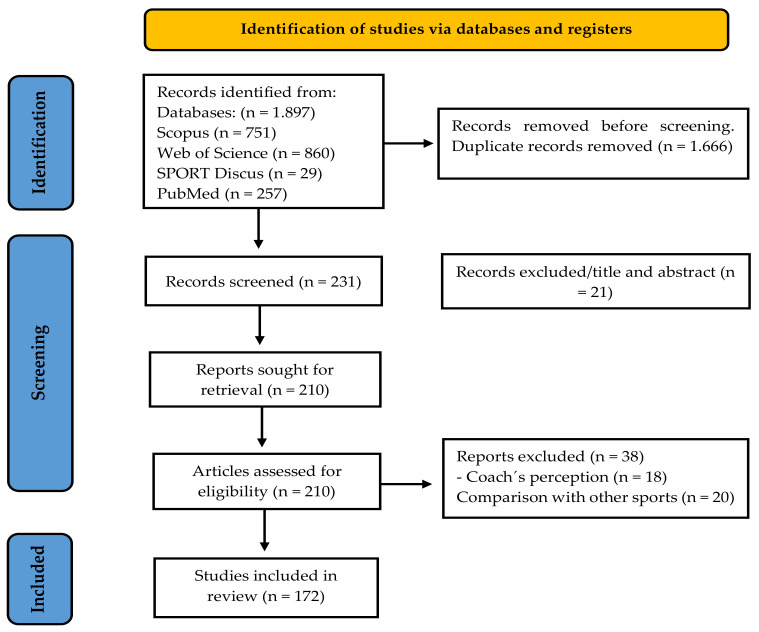
Flow diagram for the selection of studies according to PRISMA guidelines.

The search for documents was carried out on the Web of Science (WoS) database, especially in the WoS Core Collection. This database was selected because many bibliometric football-related reviews have been conducted there [50,51,52,53,54]. This data platform allows access to a large amount of information from the selected manuscripts and analyzes the influence in the scientific field [48]. Similarly, a search was carried out in PubMed, Scopus database, and SPORTDiscus. When screening the information, 88% of the documents were duplicates. Therefore, it was decided to consider only WoS studies and proceed to add documents that were not present in the other databases consulted, following the guidelines of other bibliometric studies [55,56]. A review based on the WoS database was carried out using the following terms and following the participants, interventions, and outcomes (PIO) strategy: (soccer OR football OR “team sports”) AND (knowledge OR intelligence OR “decision making” or “game performance”) AND (tactic* OR cognitive OR declarative). Finally, the search equation used was decision-making AND soccer AND players. The search was carried out on July 15 by two leading researchers (J.D.P.-U. and B.A.B.-P.). A systematic process was developed for selecting the included documents based on bibliometric criteria, such as duplication and a direct relationship with the study’s objective [57]. Initially, the 1.868 documents submitted to the metadata regulation process were analyzed to eliminate duplicates, lack full access, and no relationships to the study’s subject. After searching for documents, 172 documents that met the eligibility criteria established for this study remained (Figure 1).

### 2.3. Eligibility Criteria

The inclusion criteria of the studies were as follows: (i) focused on the study of decision-making in child soccer players, adolescents, or adults; (ii) focused on sports samples that aimed to improve training or competition; (iii) were original full-text studies reviewed by academic peers; (iv) focused on studies in soccer players or that included both genders in the study; (v) had access to the complete document to complement the first review; (vi) published between 1 January 2010 and 15 July 2024; and (vii) indexed the journal in the Journal Citation Report (JCR) in the following quartiles: Q1, Q2, Q3, and Q4. The exclusion criteria were as follows: (1) studies that considered the evaluation of team sports where soccer was included; (2) studies that considered the evaluation of robot soccer; (3) studies in sports; and (4) decision-making for the return after injury.

To select the documents, an analysis matrix was developed in Microsoft Excel based on the following categories: (1) year of publication; (2) type of document; (3) most cited articles; (4) name of authors; (5) number of authors by study; (6) keywords; (7) journals and references cited; (8) publishing groups; (9) areas of knowledge; (10) language; (11) countries; and (12) organizations.

### 2.4. Data Analysis

The identified data were extracted in two different formats: plain text and Excel. The Excel document allows descriptive and percentage analysis of the results via a Microsoft Excel spreadsheet (v. 2006, Microsoft Corporation, Redmond, WA, USA). The plain text format allows the development of analysis by coauthorship for authors, coauthorship by organizations, occurrence by keywords, citation by documents, citation by journals, citation by countries, and citation by authors with the VOSviewer program (v.6.19., Center for Science and Technology Studies, Netherlands). A fragmentation analysis was performed with a value of 2 for attraction and 1 for repulsion. The VOSviewer program allows for the creation of two-dimensional graphs [58] from nodes to establish the central relationships developed from the scientific production stored in the different databases [59].

The following laws were considered to support the bibliometric analysis: (1) Price’s law from the coefficient of determination (*R*^2^) coefficient to determine the strength of the linear relationship between two variables [60]; in this case, the number of published documents and citations over the years, as well as the number of citations. (2) Lotka’s law allows for the identification of the authors who have published the most studies [61,62], and (3) Zipf’s law is oriented to establish the occurrence of the most used keywords [63].

Similarly, the *H*-index was used, which determines academic productivity according to the number of times a document has been cited at least a minimum number of times [64].

## 3. Results and Discussion

### 3.1. Evolution of the Number of Documents

**Figure 2:**
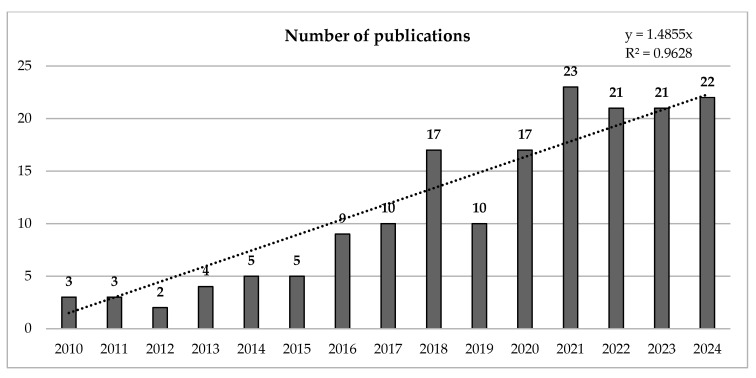
Evolution of the number of annual publications.

The increase in scientific production related to the number of documents published per year between 2010 and 2024 reveals a systematic increase in the number of published studies, with 2023 being the year with the highest production. Similarly, Figure 2 shows that in the middle of 2024, the trend reflects an increase in the number of documents. In terms of the R^2^ coefficient of the sample obtained, a substantial increase of 633.33% was identified between the first reference year (2010) and the last reference year (2024).

**Figure 3:**
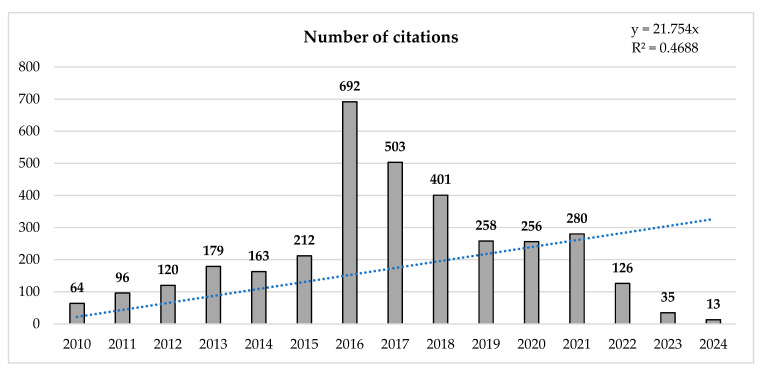
Evolution of the number of annual citations.

The number of citations per year slightly increased, and until 2016, there was a significant change of 981.25% compared with the first year (2010). The trend reveals low citation rates for 2017, 2019, and 2022, with decreases of −27.31%, −35.66%, and −55.00%, respectively, compared with 2016, 2018, and 2021 (Figure 3).

### 3.2. Types of Documents

Different types of documents have been published to socialize studies related to decision-making in football. However, the trend reveals that the most significant number is articles, which account for more than one-third of the total scientific production. In turn, few review studies exist. Research related to the type of documents that are preferably used for the socialization of the research results is empirical research. The low number of review studies for bibliometric analysis is also associated with the scientific development of specific topics (Table 1).

### 3.3. Analysis of the Most Cited Documents

**Figure 4:**
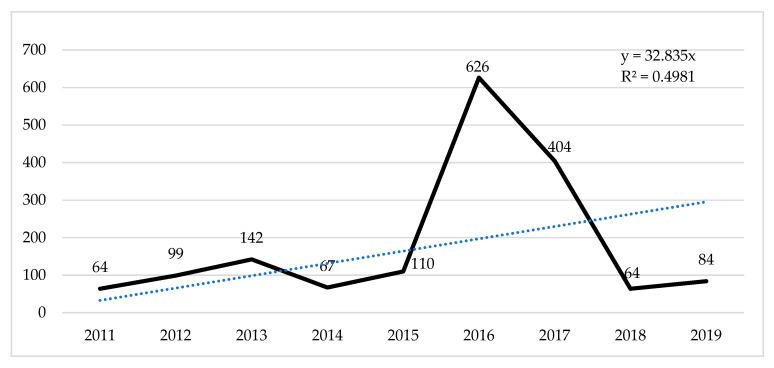
Evolution of the number of citations received per year for the most cited studies.

Figure 4 details the behavior of the total number of appointments over nine years. Given the number of documents published per year, for 2016 and 2017, it is also determined as the period that receives the most significant number of citations. The low level of citations in 2018 and 2019 for this type of document is striking, with significant decreases of −89.77 and −79.20 compared with 2016 and 2017, respectively.

The analysis of the documents that have received at least 50 citations reveals ten studies (Table 2), of which two are reviewed and the other eight are research articles. Among the ten studies included, only two considered the analysis of physiological, physical, tactical, and technical variables in the decision-making approach of soccer players. All the documents date back to far less than 2017, and in turn, six of these studies were published in journals indexed in the Journal Citation Report (JCR) in the first quartile (Q1). Within the effects that are evaluated, two significant fields can be deduced: one related to the analysis of the factors associated with perceptual–cognitive skills, mental fatigue, anticipation, creativity, and memory, whereas the second is more related to the study that the decision-making in the manifestations of specific performance of the game, between experts and novices, in the precision of technical actions, such as the pass, as well as in a methodology for the selection of athletes. Notably, of the most cited documents, two are reviews [65,66], one is a randomized controlled trial [67], one is a cross-sectional study [68], and, finally, six are experimental studies [7,69,70,71,72,73].

**Figure 5:**
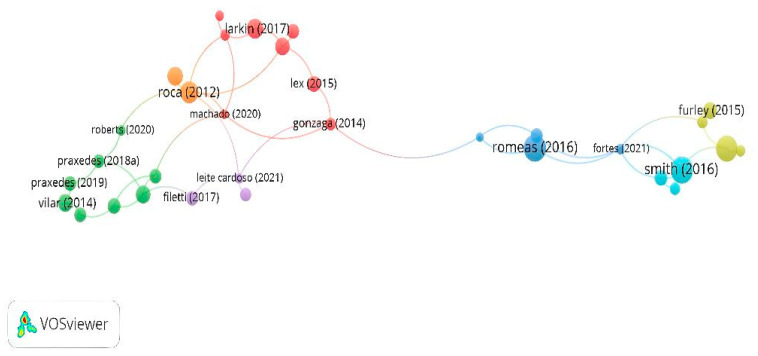
Citation of documents.

One hundred seventy-two documents included in this study were identified; thus, to analyze the citations by documents, it was established that each document was cited at least 20 times. Only 48 documents exceeded the requested threshold; only 32 seemed connected. There are seven nodes where the most cited documents are those developed by Roca et al. [70], Romeas et al. [69], and Smith et al. [67] (Figure 5).

### 3.4. H-Index

**Figure 6:**
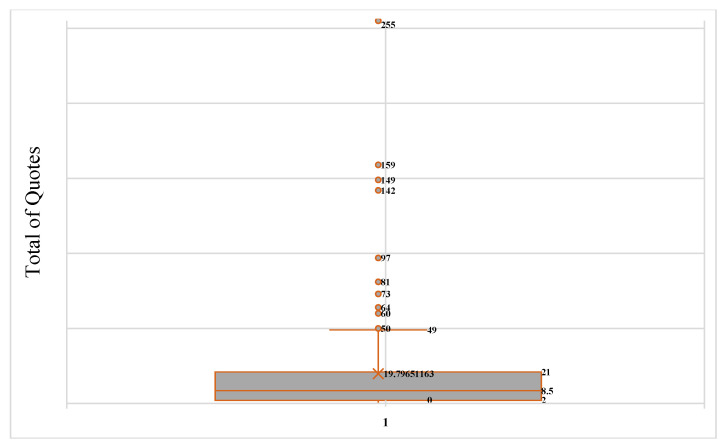
Number of citations as a function of the *H*-index.

The average number of citations per document was 19.79 (Figure 6).

### 3.5. Author Analysis

The academic cooperation evaluated in response to the number of authors shows that the most significant number of documents, close to half of the total scientific production, is between three and four authors. Moreover, the scientific production developed by six authors is greater than that carried out by one or two authors, which demonstrates the interest in favoring academic cooperation in search of the production of knowledge in the study of decision-making in football (Table 3).

**Figure 7:**
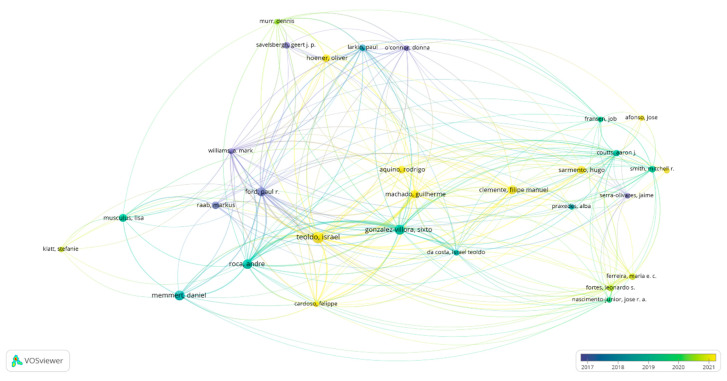
Citation by authors.

Figure 7 shows the interactions produced between the citations by authors. Five hundred eighty-one authors were established, and only thirty authors have developed three studies with a minimum of 10 citations. The size of the nodes represents the number of documents published, and the color corresponds to the connections established in response to time. There was an intense concentration between the years 2018 and 2019 led by Gonzalez-Víllora, S., while between the years 2020 and 2021, a more significant number of networks were observed, with Teoldo, I., the one that received the highest number of citations in recent years.

### 3.6. Keyword Analysis

**Figure 8:**
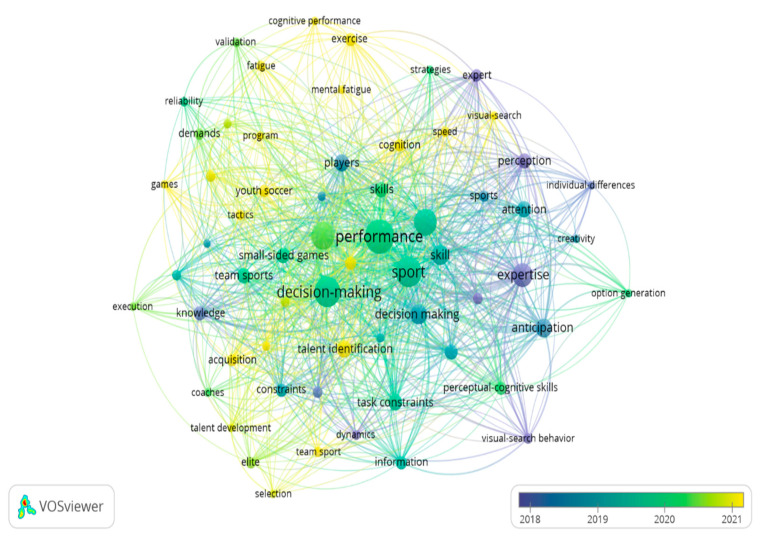
Occurrence by keywords in response to temporality.

For keyword occurrence, the word was included at least five times in each study. Thus, of the 807 words identified, only 62 met the threshold. The analysis of the most recurrent keywords refers to perceptual processes, anticipation, expertise, and performance. The most referenced concepts were “Anticipation”, “Attention”,” “Knowledge”, “Decision Making”, and “Skill”. Between 2019 and 2020, the concepts used were “performance”, “sport”, and “decision-making” (Figure 8).

Finally, between 2020 and 2021, these most referenced concepts were “Talent Identification”, “Mental Fatigue”, “Cognition”, “Visual Search”, and “Demands”. Thus, the most recent concepts are associated with competition evaluation processes, the development of programs related to game tactics, and the recognition of coaches’ perceptions (Figure 8).

**Figure 9:**
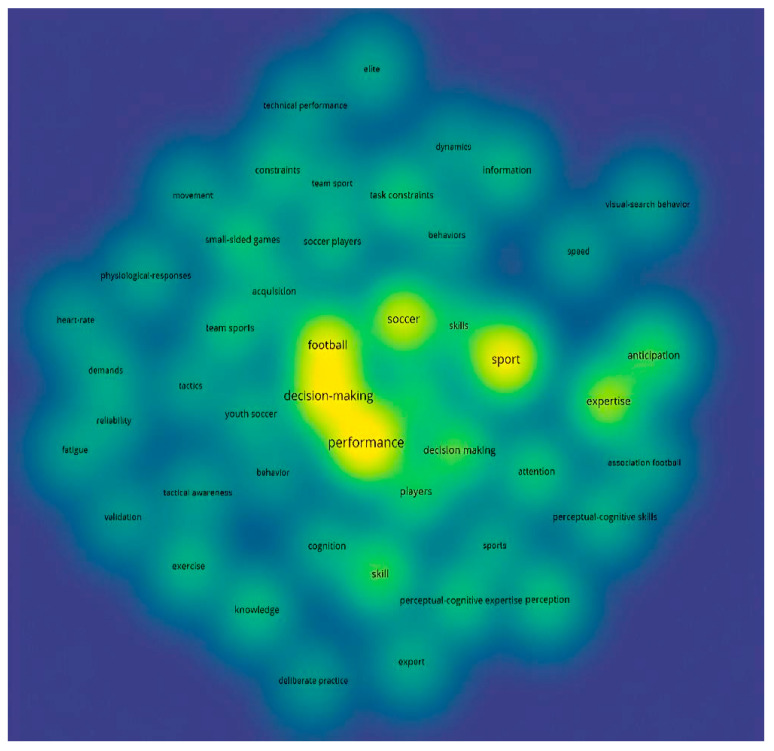
Occurrences by keywords in response to density.

Moreover, according to the density map in Figure 9, the concepts related to the number of times that have been cited the most are “*Performance*” (*n* = 68), “*Sport*” (*n* = 55), “*Decision-Making*” (*n* = 54), “*Football*” (*n* = 45), “*Soccer*” (*n =* 41), “*Expertise*” (*n* = 32), “*Skill*” (*n* = 23), and “*Anticipation*” (*n* = 22).

### 3.7. Analysis of Cited Journals and References

**Figure 10:**
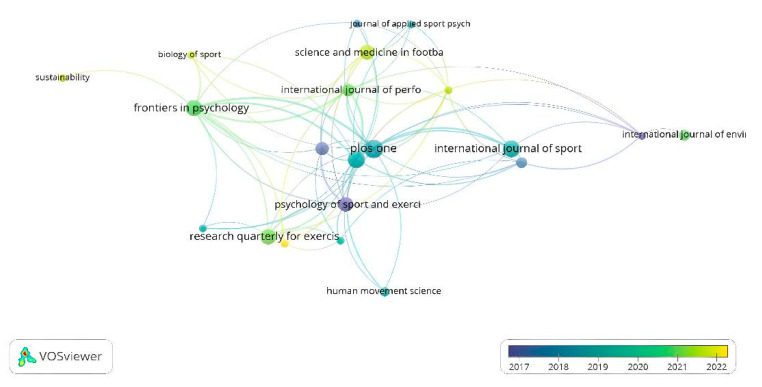
Citations by journal.

Seventy-seven journals published the 172 documents found for this bibliometric review. To analyze the citations by journals, the journal was considered to have published at least two documents and received at least ten citations. Thus, only 21 magazines met the threshold. Similarly, in the citation map by journal, the journals with the most significant number of published documents are the *Journal of Sport Sciences* (ten documents and 437 citations), *PLoS ONE* (eleven documents and 349 citations), and *Psychology of Sport and Exercise* (seven documents and 259 citations) (Figure 10).

**Figure 11:**
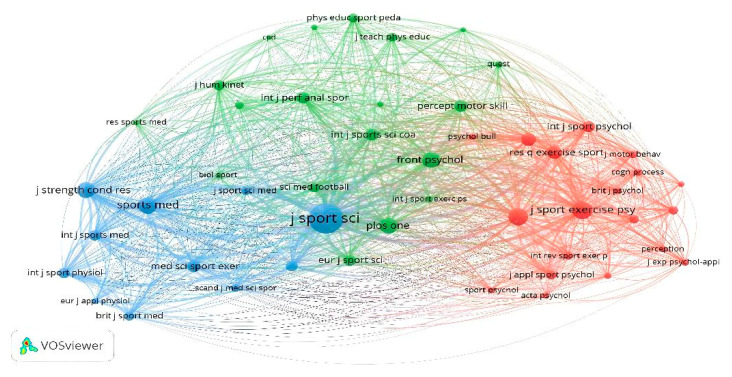
Cocitations by journals.

Similarly, for the analysis of the cocitations between the journals, 1921 sources were found, where it was established that the minimum number of citations for each journal was 20. Only 49 journals met the requested threshold (Figure 11). The journals with the highest number of citations are the *Journal of Sport Sciences* (*n* = 615), the *Journal of Sport and Exercise Psychology* (*n* = 220), *Psychology of Sport and Exercise* (*n* = 206), and *Sports Medicine* (*n* = 189).

**Figure 12:**
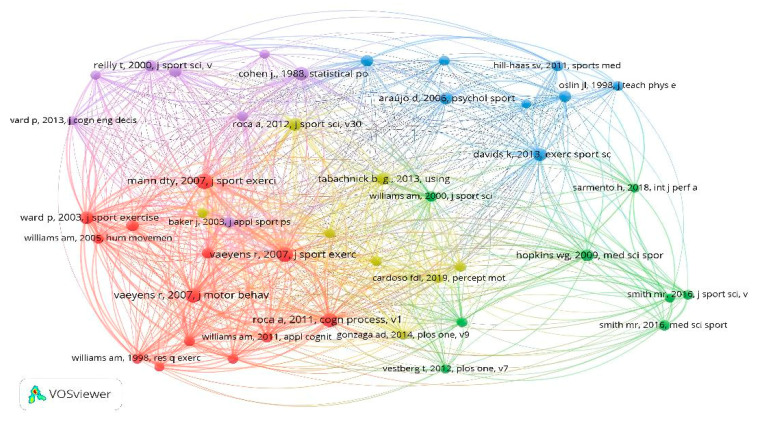
Nodes by cited references.

The analysis of the cited references reveals that there are 5251, 45 of which have been cited at least ten times. These methods were developed by Mann et al. [74], Vaeyens et al. [75], and Roca et al. [76] (Figure 12).

### 3.8. Analysis of Publishing Groups

The scientific production by the publishers that publish scientific knowledge on the study of decision-making in football reveals how Taylor & Francis is the publishing group that publishes the most significant number of documents and, in turn, receives the most significant number of citations. It also details how five publishers have received more than 300 citations (Taylor & Francis, Elsevier, Springer, Public Library Science, Human Kinetics). The first four publishers represent half of the total scientific production (51.74%), and in turn, four publishers have received more than 1000 citations (Table 4).

### 3.9. Areas of Knowledge

The analysis of the areas of knowledge where the documents on the study of decision-making in football are published reveals that the most prolific and cited areas are hospitality, leisure, sport, and tourism, with an average of 15.40; sport sciences, with 21.89; and multidisciplinary sciences, with 50.33 citations per published document. Similarly, the first two areas cover more than half of the published documents (55.81%), which reflects the scientific community’s interest in developing this type of study associated with specific areas (Table 5).

### 3.10. Language and Country Analysis

**Figure 13:**
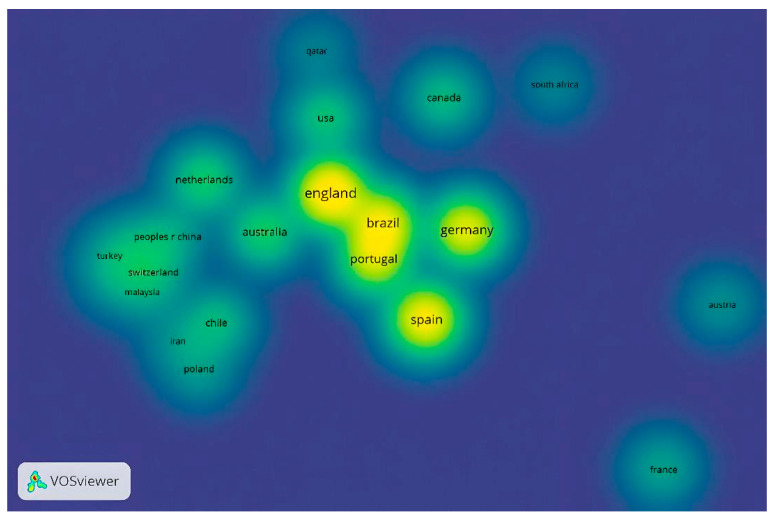
Citation by country.

The analysis by language shows that the most significant production is generated in English (*n* = 166), with 96.51% of the total scientific production, followed by Spanish (*n* = 5), with 2.90%, and Portuguese. In addition, among the 172 documents reviewed and included in this bibliometric study, only one study that was different from English and Spanish (Portuguese) was found. Figure 13 establishes the citations of documents by country. Only 40 countries have contributed to the knowledge of decision-making. To establish the threshold, each country was determined to have published at least two studies and received ten citations.

Thus, only 22 countries met the threshold. The countries that receive the greatest number of documents and citations are England (*n* = 46 documents and 996 citations), Germany (*n* = 32 documents and 749 citations), Spain (*n* = 38 documents and 597 citations), Portugal (*n* = 27 documents and 481 citations), and Brazil (*n* = 34 documents and 395 appointments). Specifically, several European countries (Poland, Switzerland, Turkey, Netherlands), Asian countries (China, Malaysia, Iran, Qatar), Africa (South Africa), South America (Chile), North America (Canada, United States), and Oceania (Australia) are increasing their scientific production in the study of decision-making in football. Finally, there are countries on each of the continents, which highlights the importance of this theme among the scientific–academic community.

### 3.11. Organizations

**Figure 14:**
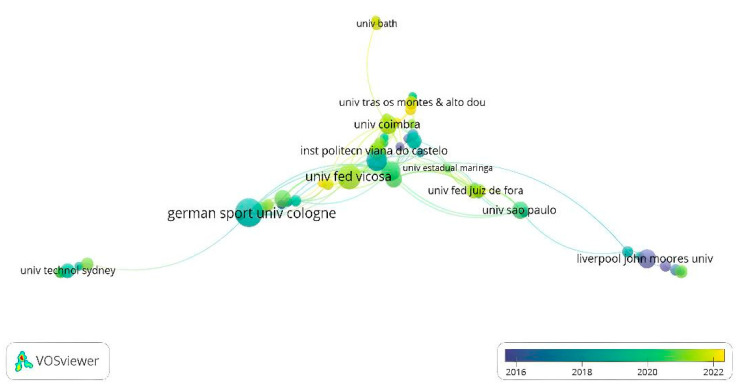
Coauthorship by organizations.

It is recognized that 313 organizations have been studying decision-making in football. Only 60 organizations have published at least two studies and have received at least ten citations. Specifically, German Sport University Cologne is the organization that has the most publications and citations (*n* = 19 and 531). Owing to the weather (2016–2018), there is a strong trend among European universities, among which German Sport University Cologne and Liverpool John Moores University stand out. Between 2020 and 2022, there was a strong trend of Brazilian and Portuguese universities, of which the following stand out: the Federal University of Viçosa, the University of São Paulo, the University of Coimbra, and Juiz de Fora Federal University (Figure 14).

## 4. Discussion

### 4.1. Main Findings by Bibliometric Criteria

To our knowledge, this is the first study that performs a bibliometric analysis on scientific publications related to decision-making in soccer. Therefore, by analyzing the evolution and trend of the number of studies related to decision-making in soccer in the last 15 years, the following findings were determined. (1) A total of 172 documents related to the study of decision-making were published between 2010 and July 2024. (2) The number of studies increased by 633.33% between 2010 and 2024, and, in turn, the years with the highest production were between 2021 and 2024. (3) There is a preference for developing research articles (89.53%), with a small percentage of review studies. The years 2016 and 2017 received the most appointments. Documents that have received at least 50 citations are preferably research articles and have been published in Q1 journals. From 2010 to 2024, only two review studies were reported, and no meta-analytical study, scoping review, or bibliometric analysis was reported. At least ten studies have received 50 appointments. (4) The average number of citations per document was 19.79. (5) Half of the total scientific production was carried out by between three and four authors (44.76%). Five hundred eighty-one authors were established; only thirty authors have developed three studies and have a minimum of 10 citations. Between 2018 and 2019, scientific production was led by Gonzalez-Villora, S., whereas between the years 2020 and 2021, there was a greater number of networks. Teoldo, I. is the one who has received the highest number of citations in recent times. (6) The most recent concepts are associated with competition evaluation processes, the development of programs related to game tactics, and the recognition of coaches’ perceptions. These are performance, sport, decision-making, football, soccer, expertise, skill, and anticipation. (7) A total of 77 journals published the 172 documents identified in this bibliometric review. The journals that have published the greatest number of documents are the *Journal of Sport Sciences*, *PLoS One*, and *Psychology of Sport and Exercise*. For the analysis of the cocitations between the journals, 1921 sources were found, so the journals with the highest number of citations are the *Journal of Sport Sciences*, the *Journal of Sport and Exercise Psychology*, *Psychology of Sport and Exercise*, and *Sports Medicine*. (8) Taylor & Francis is the publishing group that has published the most documents and received the most citations. (9) The most developed areas, according to the WoS Core, are first, hospitality, leisure, sport, and tourism, and second, sport sciences. (10) Studies on decision-making in soccer have been published in three different languages, with English being the predominant language. (11) A total of 313 organizations have been studying decision-making in football. Only 60 organizations have published at least two studies and have received at least ten citations. The leading institutions are the German Sport University Cologne, Liverpool John Moores University, the Federal University of Viçosa, the University of São Paulo, the University of Coimbra, and the Juiz de Fora Federal University. (12) The evaluated sample mainly consists of studies of professional players, with a low percentage of studies of children (6–12 years) and women’s soccer.

### 4.2. Some Considerations from Major Studies

The increase in studies in specific time windows that study football via bibliometric analyses reveals a preference for the study of research articles over review studies. This is what is detailed in the study developed by García-Angulo and Ortega [77] when studying the scientific production of the soccer goalkeeper. These results are associated with other bibliometric studies that demonstrate similar findings in the studies of small-sided games (SSGs) in soccer [54], youth soccer [52], biomechanics in soccer [78], and research trends and future directions of women’s football [79] when the studies between 2010 and 2023 are analyzed. This demonstrates the interest of different professionals in producing knowledge about the study of soccer.

Another of the main findings shows that the number of publications has gradually increased, especially in empirical research studies. In contrast, the number of review studies does not exceed 10% of the total production, like those found in other bibliometric studies on plyometrics in sport [80] and relative age [81]. In this way, the results reported by this bibliometric analysis can be used by different researchers to develop research in less explored themes and fields of study, even more so when the existence of any study related to the development of meta-analytical reviews or scoping reviews on decision-making in football is reported. In contrast, there are different scoping reviews on existing tests to evaluate knowledge and tactical performance in soccer players [82], revealing that there are approximately 29 tests/instruments that allow for the evaluation of the tactical domain beyond the investigative approach and the method of information collection.

A bibliometric study analyzing the 100 most cited articles on sports medicine and exercise revealed that the two most studied anatomical areas were the knee and the brain. At the same time, a narrative review was the most common study [55]. This demonstrates the importance of specificity when studying different areas of knowledge since, for the analysis of decision-making in soccer, the two most studied themes are performance, expertise, and skill. The contribution of bibliometric analyses implies the importance of the findings presented in this type of study because they offer a global overview of the state of the scientific evidence [83].

The studies of the most cited articles also reveal the use of technology [13] for monitoring, and the results are associated with determining the influence of physical, physiological, technical, and tactical variables [71,84]. Among these relationships, game performance is associated with technical–tactical skills (e.g., passing, ball driving, marking, tackling, and intercepting) and decision-making adapted to the context of the game situation [68]. Likewise, the two investigative tendencies found in the study of decision-making in football establish, on the one hand, the study of perceptual–cognitive skills [85], such as mental fatigue, creativity, work memory, and anticipation; on the other hand, the study of decision-making in the technical–tactical performance of athletes is evident, through precision, the factors that condition the pass [86], tactical awareness [87], and tactical decision-making [88].

In the same way as the two investigative trends identified, the study developed by Teoldo et al. [89] relates to fatigue and visual peripheral perception, demonstrating that physical fatigue is conditioning when making faster decisions in association with the tactical actions that players must execute and that, in essence, physical fatigue influences only the response time to make decisions. In this sense, the influence of peripheral perception, physical performance, and tactical behavior in soccer players has been defined so that acute physical fatigue does not influence peripheral perception; however, both physical performance and tactical behaviors are diminished, causing errors in defensive movements and a decrease in offensive tactical actions close to the ball [90]. Moreover, a study by de Souza Fortes et al. [91] revealed that the mental fatigue produced by playing video games before competition affects decision-making in professional players.

By this, studies on the effects of fatigue on performance [92] demonstrate a tendency toward the use of objective indicators for its evaluation, demonstrating that stressful environments produced by heat or stress tend to increase the likelihood that athletes make mistakes in decision-making in situations where they commonly do not perform them [93]. Another systematic and meta-analytical review on the effects of mental fatigue on career performance and tactical behavior concluded that mental fatigue has no effect on the total running distance and tactical behavior expressed by players in the SSG [94]. This is perhaps one of the main contributions of this bibliometric review study because it helps to favor the current panorama of research trends from keywords, the analysis of the most cited studies, the countries that develop the most studies, and the organizations/institutions and authors that have been leading scientific production. With this, it is concluded that there are physical capacities (asymmetries, changes in direction, jumps, accelerations, decelerations), sex (female), population groups (children’s soccer, youth soccer), countries of the continents (South American, Asian, African), and concepts that need more excellent research development. All of this is because sports performance is a process through which athletes develop specific psychological behaviors and activities [95,96].

These results highlight the importance of understanding decision-making from different perspectives, so more research is needed to determine the effect and influence of the moment of the competition, the result, the local or visitor play, the title or emergence, age, and sex, among other factors. However, this type of specific knowledge provided by different studies must be adapted to the specific needs of those who guide the preparation process and the transfer of this scientific knowledge to a practical and daily task of the coach and the athlete [97]. Thus, the main gaps in the literature such as greater international cooperation and further development of studies on women’s soccer, youth soccer, and children’s soccer will allow the establishment of lines of research that focus on the study of decision-making in different contexts of training and competition from observational studies, case studies, and longitudinal studies. It is also necessary to further develop qualitative studies that help us to understand the perceptions of decision-making from the perspective of the coach–athlete.

### 4.3. Emerging Concepts and Instruments Used for the Evaluation of Decision-Making

Finally, it has been confirmed that a study that deepens a theme can expand the research horizons, revealing that there are emerging concepts associated with decision-making and the identification and selection of sports talent. Among them are “*Constraints*”, “*Acquisition*”, “*Visual-Search*”, “*Program*”, “*Cognitive performance*”, and “*Tactics*”. To carry out different studies related to the evaluation of decision-making in soccer players of different ages, there are scientifically validated technological tools that allow for the evaluation of performance. Among them is the soccer tactical evaluation system (FUT-SAT) that allows for the analysis of the fundamental principles of the game [98]. Other instruments that can be used are the Game Performance Assessment Instrument (GPAI) that allows for the evaluation of four specific domains (game performance, support, decision-making, skill execution) [99], the Performance Assessment in Team Sports (TSAP) [100], the Game Performance Evaluation Tool (GPET) [101], and the TacticUP Video Test for Soccer [13].

### 4.4. Future Perspectives

The consideration of decision-making as a study theme has increased the research that has been developed to understand soccer’s technical–tactical behavior. However, very few studies with multivariate designs allow us to understand the relationships established between decision-making and other skills, characteristics, and processes. Thus, future studies could explore this panorama and provide more significant contributions to the influence that decision-making has on players’ sports performance, health, and well being.

Given the future perspectives of studies focused on evaluating, identifying, and recognizing the contributions of the study of decision-making, specifically on perceptual–cognitive skills and technical–tactical components in different population samples, the need arises to carry out a more significant number of review studies, including meta-analyses, scoping reviews, and more specific bibliometric analyses. Similarly, in evaluating athletes, it is necessary to develop a greater number of randomized controlled trials, longitudinal designs, and case studies that provide a greater understanding of the effects of decision-making on other physical, technical, tactical, physiological, psychological capacities, etc.

### 4.5. Limitations

This study presents different limitations associated with the heterogeneity of population samples and evaluated characteristics, the denomination with which decision-making is studied, and the variables that are related. Another bias of the present study was that only the review of documents in primary databases such as WoS, PubMed, Scopus, and SPORTDiscus were considered; therefore, it is recommended that future bibliometric studies on this topic review the information in other databases to obtain a deeper understanding of the topic under study. In addition, other limitations arise, and to reduce bias, future research should consider the analysis of the language of publication, open-access documents, searching in other databases, and using other search concepts to study decision-making.

## 5. Conclusions

Literature specializing in the study of decision-making in football prefers the study of technical and tactical variables and, to a lesser extent, physical and physiological variables. By studying the most cited studies, it is confirmed that empirical studies and very few review studies prevail. There is a strong tendency of South American authors and organizations/institutions, mainly Brazilians, to study decision-making. Two critical approaches have been carried out in the study of decision-making: one is related to the perceptual–cognitive skills evaluated in the laboratory and the field through working memory, creativity, and mental fatigue, whereas the second approach is more associated with the manifestation of the technical–tactical behaviors of the players, which are evaluated from the use of video or tests on tactical knowledge. By studying the terms used for the study of decision-making, the investigative trends related to sports performance are oriented as follows: “*Talent identification*”, “*Youth Soccer*”, “*Games*”, “*Coaches*”, “*Cognition*”, “*Speed*”, “*Validation*”, “*Reliability*”, and “*Program*”. Research trends suggest that the study of decision-making should be addressed through interdisciplinary approaches or applied in sport training contexts. On the other hand, the central countries that have been leading in scientific production are also the countries with the most important leagues, among which the following stand out: “England”, “Spain”, “Germany”, “Portugal”, and “Brazil”. This highlights the importance of interdisciplinary and transdisciplinary research in the study of decision-making in soccer.

The scientific evidence must allow us to glimpse the effects of different training protocols on the induced adaptations for samples identified in the present study as female soccer athletes. In the context of soccer, this type of study could help determine how decision-making helps us to understand the relationship with other variables associated with the physical and mental health of athletes at different ages and levels. The control of training load from the study of decision-making is an essential component in any training process to promote the health and well-being of all athletes. With this, the analysis of decision-making would help coaches and technical staff to consider individualized follow-ups to reduce the stress produced in competition and to think about interdisciplinary support to promote healthy habits and active lifestyles outside the training–competition spaces.

## Figures and Tables

**Table 1:** Types of documents published.

Type of Document	Number of Documents	Percentage, %
Article	154	89.53
Review	11	6.39
Meeting Abstract	5	2.90
Book Chapter	2	1.16
Total	172	100

**Table 2:** Most cited articles.

Author and Publication Year	Type	Journal	Cat Q, Cat Rank JCR, JIC Cat 2023	Effects	Physio Outc	Phys Outc	Tact Outc	Techn Outc	Main Conclusions	Tot Cit	Av Cit per Year *
Rein and Memmert (2016) [65]	Narrative review	*Springerplus*	-	Big data and soccer tactics	Yes	Yes	Yes		The introduction of big data technologies will also require discussions within the research community about how to share data and techniques across research teams. Future soccer research will have to embrace a more robust multidisciplinary approach.	255	31.87
Smith et al. (2016) [67]	Article—randomized controlled Trial	*Journal of Sports Sciences*	Q1 31/127 Sport Sciences	Mental fatigue	Yes		Yes		Mental fatigue had unclear effects on most visual search behavior variables. The results suggest that mental fatigue impairs the accuracy and speed of soccer-specific decision-making. These impairments are unlikely to be related to changes in visual search behavior.	149	18.62
Romeas et al. (2016) [69]	Article—experimental design	*Psychology of Sport and Exercise*	Q1 21/127 Sport Sciences	Precision in passing decision-making			Yes	Yes	Training to process complex and dynamic visual scenes has not only revealed superior learning ability in soccer players but has also improved their passing decision-making accuracy in the field.	149	18.62
Roca et al. (2012) [70]	Article—experimental design	*Journal of Sports Sciences*	Q1 31/127 Sport Sciences	Acquisition of superior anticipation			Yes	Yes	The average hours accumulated per year during childhood in soccer-specific play activity were the strongest predictor of perceptual–cognitive expertise.	142	11.83
Roca et al. (2013) [7]	Article—experimental design	*Journal of Sport and Exercise Psychology*	Q2 59/114 Psychology, Applied	Perceptual–cognitive skills			Yes	Yes	Skilled players reported more accurate anticipation and decision-making than less skilled players, and their superior performance was underpinned by differences in task-specific search behaviors and thought processes.	99	9.00
Gonçalves et al. (2017) [71]	Article—experimental design	*Journal of Strength and Conditioning Research*	Q1 9/127 Sport Sciences	Restrictions on tactical behavior, physical, and physiological performances	Yes	Yes	Yes	Yes	The effects of limiting players’ spatial exploration greatly impaired the coadaptation between teammates’ positioning while decreasing their physical and physiological performances. These results allow for a better understanding of players’ decision-making processes according to specific task rules.	97	13.85
Qader et al. (2017) [66]	Review	*Measurement*	Q1 17/180 Engineering, Multidisciplinary	Methodology for football player selection	Yes	Yes	Yes	Yes	Three main groups of tests were utilized to assess the players: fitness, skills, and anthropometrics. This study improved the understanding of the measures as criteria for player selection. TOPSIS is an effective tool used to solve player selection problems.	67	9.57
Diaz del Campo et al. (2011) [68]	Article—cross-sectional study	*Perceptual and Motor Skills*	Q4 86/99 Psychology, Experimental	Differences between experts and novices			Yes	Yes	Expert players remained superior in decision-making ability when variation in skill execution was controlled. Decision-making differences between levels of expertise decreased with age regarding the first level (skill selection) and increased with age regarding the second level (tactical–context adaptation).	64	4.92
Furley and Memmert (2015) [72]	Article—experimental design	*Frontiers in Psychology*	Q2 62/218 Psychology, Multidisciplinary	Creativity and working memory			Yes	Yes	The pattern of results provided evidence that domain–general working memory capacity is not associated with creativity in a soccer-specific creativity task. Future research on the role of working memory in everyday creative performance needs to distinguish between different types of creative performance while also taking the role of domain-specific experience into account.	60	6.66
Lex et al. (2015) [73]	Article—experimental design	*PLoS One*	Q1 33/135 Multidisciplinary Sciences	Cognitive representations and cognitive processes			Yes	Yes	In contrast to less experienced soccer players, more experienced soccer players possess a functionally organized cognitive representation of team-specific tactics. Moreover, the more experienced soccer players reacted faster to tactical decisions because they needed fewer fixations of similar duration.	50	5.55

Note: The average number of citations per year was calculated from the date of publication to 8 July 2024 *. Cat Q: Category Quartile; Cat Rank JCR: Category Rank in Journal Citation Reports; JIC Cat 2023: Journal Indicator Citation 2023; Physio Outc: Physiological Outcomes; Phys Outc: Physical Outcomes; Tact Outc: Tactical Outcomes; Techn Outc: Technical Outcomes; Tot Cit: Total Citations; Av Cit per year: Average Citation per year; TOPSIS: technique for order performance by similarity to ideal solution.

**Table 3:** Total number of documents per author.

Number of Authors	Number of Studies	Percentage, %
1–2	28	16.27
3–4	77	44.76
5–6	44	25.58
7–8	17	9.88
≥9	6	3.48

**Table 4:** Total number of documents published by the editorial groups.

Name	Number of Documents	Number of Citations
Taylor & Francis	42	926
Elsevier	19	454
Frontiers Media SA	15	140
Multidisciplinary Digital Publishing Institute (MDPI)	13	65
Springer	11	407
Public Library Science	11	349
Sage Publications Ltd.a.	11	164
Human Kinetics Publ INC	8	325
Federación Española Asociación Docentes Educación Física	6	7
Termedia Publishing House Ltd.a.	3	24
Total: 10 editorials	139/172	2861

**Table 5:** Areas of knowledge according to Web of Science categories.

Areas *	Number of Publications	Number of Citations
Hospitality, Leisure, Sport, and Tourism	49	755
Sport Sciences	47	1029
Psychology	23	403
Multidisciplinary Sciences	12	604
Neurosciences	10	96
5 Areas	141/172	2887

Note: * The same item may be considered in more than one area.

## Data Availability

Data supporting the findings of this study are available from the corresponding author upon reasonable request.
